# Mitochondria sustain store-operated currents in colon cancer cells but not in normal colonic cells: reversal by non-steroidal anti-inflammatory drugs

**DOI:** 10.18632/oncotarget.19430

**Published:** 2017-07-21

**Authors:** Miriam Hernández-Morales, Diego Sobradillo, Ruth A. Valero, Eva Muñoz, Daniel Ubierna, Mary P. Moyer, Lucía Núñez, Carlos Villalobos

**Affiliations:** ^1^ Institute of Molecular Biology and Genetics (IBGM), Spanish National Research Council (CSIC), Valladolid, Spain; ^2^ INCELL Corporation, San Antonio, TX, USA; ^3^ Department of Biochemistry and Molecular Biology and Physiology, University of Valladolid, Valladolid, Spain

**Keywords:** store-operated Ca^2+^ entry, store-operated currents, mitochondria, colorectal cancer, non-steroidal anti-inflammatory drugs

## Abstract

Tumor cells undergo a critical remodeling of intracellular Ca^2+^ homeostasis that contribute to important cancer hallmarks. Store-operated Ca^2+^ entry (SOCE), a Ca^2+^ entry pathway modulated by mitochondria, is dramatically enhanced in colon cancer cells. In addition, most cancer cells display the Warburg effect, a metabolic switch from mitochondrial metabolism to glycolysis that provides survival advantages. Accordingly, we investigated mitochondria control of store-operated currents (SOCs) in two cell lines previously selected for representing human normal colonic cells and colon cancer cells. We found that, in normal cells, mitochondria are important for SOCs activity but they are unable to prevent current inactivation. In contrast, in colon cancer cells, mitochondria are dispensable for SOCs activation but are able to prevent the slow, Ca^2+^-dependent inactivation of SOCs. This effect is associated to increased ability of tumor cell mitochondria to take up Ca^2+^ due to increased mitochondrial potential (ΔΨ) linked to the Warburg effect. Consistently with this view, selected non-steroidal anti-inflammatory drugs (NSAIDs) depolarize mitochondria, inhibit mitochondrial Ca^2+^ uptake and promote SOC inactivation, leading to inhibition of both SOCE and cancer cell proliferation. Thus, mitochondria sustain store-operated currents in colon cancer cells but not in normal colonic cells and this effect is counteracted by selected NSAIDs providing a mechanism for cancer chemoprevention.

## INTRODUCTION

The Warburg effect, first reported by Otto Warburg [1], is an aberrant metabolic profile of most tumors characterized by a high glycolytic rate, despite the abundance of O_2_. The Warburg effect confers numerous advantages to tumor cells, including enhanced proliferation, invasion and cell death resistance [2]. The mechanism underlying this effect is defective mitochondrial ATP synthesis associated to H^+^-ATP synthase dysfunction [3], resulting in enhanced anaerobic glycolysis. Another consequence of defective H^+^-ATP synthase activity that has not been addressed is the possible influence of the changes in mitochondrial potential (ΔΨ) to intracellular Ca^2+^ homeostasis. Intracellular Ca^2+^ is involved in several cell physiological processes including those underlying cancer hallmarks such as cell proliferation, migration, invasion and cell survival [4]. In addition, mitochondria are critical players in intracellular Ca^2+^ homeostasis in health and disease [5]. Mitochondria take up Ca^2+^ through the mitochondrial Ca^2+^ uniporter (MCU), a recently cloned, Ca^2+^-activated, Ca^2+^ channel located at the inner mitochondrial membrane [6, 7]. The concentration of free Ca^2+^ inside the mitochondrial matrix ([Ca^2+^]_mit_) is the same as in the cytosol in resting conditions (100 nM) [5] and, therefore, there is no chemical gradient across the inner mitochondrial membrane. However, the ΔΨ of about –180 mV results in a large driving force that enables mitochondrial Ca^2+^ uptake through the MCU [5]. Moreover, the ability of mitochondria to buffer entering Ca^2+^ may modulate the slow, Ca^2+^-dependent inactivation of Ca^2+^-release activated Ca^2+^ channels (CRAC) involved in store operated Ca^2+^ entry (SOCE) [8, 9] as well as of IP_3_ receptor channels responsible for Ca^2+^ release from intracellular stores at the endoplasmic reticulum (ER) [10].

SOCE is a ubiquitous Ca^2+^ influx pathway involved in many different cell functions in essentially all types of cells [11]. This pathway, first proposed by James W. Putney in 1986 [12], is activated after agonist-induced release of Ca^2+^ from intracellular stores at the ER by IP_3_ and/or ryanodine receptors. The decrease in ER Ca^2+^ levels is sensed by stromal interaction proteins (STIM), particularly STIM1, [13] that oligomerize and interact with plasma membrane channels Orai1 [14] and/or transient receptor potential (TRP) channels [15]. In some cell types, regulation of SOCs and SOCE by mitochondria depends on the Ca^2+^ buffering capacity of mitochondria that may prevent the slow, Ca^2+^-dependent inactivation of CRAC [8, 9]. However, whether mitochondria control equally SOCs in normal and tumor cells has not been addressed.

Using well established cell models of normal colonic (NCM460) cells and colon cancer (HT29) cells [16], we have reported recently that SOCE and the underlying store-operated currents (SOCs) are dramatically enhanced in human colon cancer cells compared with their normal counterparts [17]. Interestingly, increases in both SOCE and SOCs contributes to colon cancer hallmarks [17]. SOCE is also enhanced in a series of cancers including breast, prostate, hepatoma, glioblastoma, etc. and contribute to aberrant cell migration, invasion and survival [18]. In our established cell models, we found that differences in SOCs and SOCE are mediated, at least in part, by changes in expression of molecular players including STIM1, Orai and TRPC1 [17]. However, whether mitochondria contribute to differences in SOCE between normal colonic and colon cancer cells remains to be addressed.

We have reported that aspirin metabolite salicylate and other non-steroidal anti-inflammatory drugs (NSAIDs) may depolarize mitochondria and inhibit SOCE in different cell types including colon cancer cells [19–22]. However, the effect of mitochondrial uncouplers on SOCs in colon cancer cells relative to normal colonic cells has not been investigated. These data are relevant as overwhelming evidence suggest that aspirin and other NSAIDs may prevent colon cancer and are considered for colon cancer chemoprevention in high risk individuals [23, 24].

Accordingly, we have investigated control of SOCs by mitochondria in normal and colon cancer cells. Our results indicate that mitochondria modulate differentially SOCs in normal and colon cancer cells. In normal cells, mitochondria are critical for SOC activity but currents inactivate regardless of mitochondrial status. In contrast, in colon cancer cells, mitochondria are not required for SOC activation but are essential to prevent the slow, Ca^2+^-dependent inactivation. These effects are likely due to the larger capability of tumor cell mitochondria to take up Ca^2+^ probably because of the Warburg effect. Finally, we show that selected NSAIDs counteract the effects of the Warburg effect and promote the slow, Ca^2+^ dependent inactivation of SOCs, thus providing a mechanism for colon cancer prevention by these compounds.

## RESULTS

### Store-operated Ca^2+^ entry is larger in colon cancer cells than in normal colonic cells and differentially sensitive to FCCP

**Figure 1:**
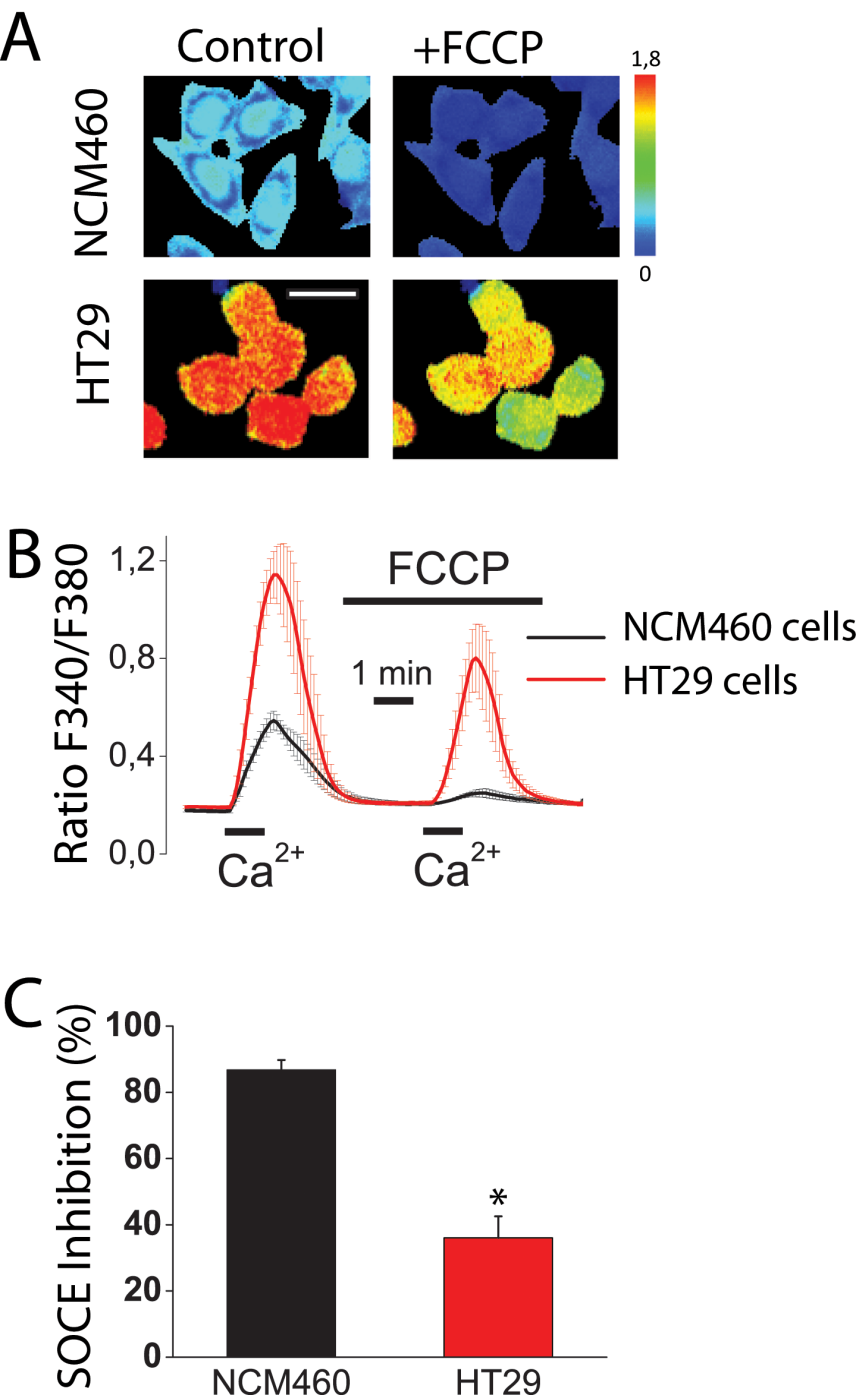
Mitochondria control store-operated Ca^2+^ entry (SOCE) in normal colonic cells and colon cancer cells Effect of mitochondrial depolarization with FCCP (10 μM) on SOCE in NCM460 and HT29 cells. Fura2-loaded cells were treated with thapsigargin (1 μM, 10 min) off the record in Ca^2+^-free medium to deplete intracellular Ca^2+^ stores. Cells were then subjected to fluorescence imaging and stimulated with Ca^2+^ containing medium to induce SOCE. (**A**) Pictures show Ca^2+^ images in pseudocolor of NMC460 and HT29 cells stimulated with extracellular Ca^2+^ in the absence (Control) or the presence of the mitochondrial uncoupler FCCP. Images correspond to maximum Ca^2+^ peaks induced by SOCE before (Control) and after (FCCP) treatment with FCCP. Bar scale correspond to 15 μm. (**B**) Traces are average Ratio F340/F380 recordings (mean ± S.E.) from 5–6 independent experiments in normal (black) and colon cancer (red) cells, respectively. (**C**) Percent inhibition of SOCE induced by FCCP (mean ± SEM) in normal (NCM460) and colon cancer cells (HT29 cells. Data are from 5–6 independent experiments, respectively. *p < 0.05.

We have investigated SOCE and the effect of mitochondrial depolarization on SOCE in normal colonic and colon cancer cells. For this end, we used NCM460 and HT29 cells that have been previously characterized as models of normal colonic and colon cancer cells, respectively [16]. Initially, role of mitochondria in SOCE was evaluated observing the effects of mitochondrial uncoupler carbonyl cyanide–4-trifluoromethoxy phenylhydrazone (FCCP) on SOCE in both normal and cancer cells. FCCP was added in the presence of oligomycin, to prevent the ATP synthase to work in reverse mode. Calcium imaging experiments show (Figure 1A) that, consistently with our previous results [17], SOCE is much larger in colon cancer cells than in normal colonic cells. Mitochondrial depolarization with FCCP inhibits significantly SOCE in tumor cells and nearly abolishes it in normal cells (Figure 1B, 1C). As reported previously in other cell types, mitochondria depolarization limits the driving force for mitochondrial Ca^2+^ uptake [8, 9], thus unmasking their contribution to SOCE. These results suggest that mitochondria may control differentially SOCE in normal colonic and colon cancer cells. However, calcium imaging permits only an indirect measure of SOCE and FCCP may have effects on plasma membrane potential. To avoid these shortcomings we investigated SOCs directly using the voltage-clamp configuration of patch-clamp electrophysiology.

### Mitochondria influence SOCs maximal amplitude in normal colonic cells but not the slow, Ca^2+^-dependent inactivation

**Figure 2:**
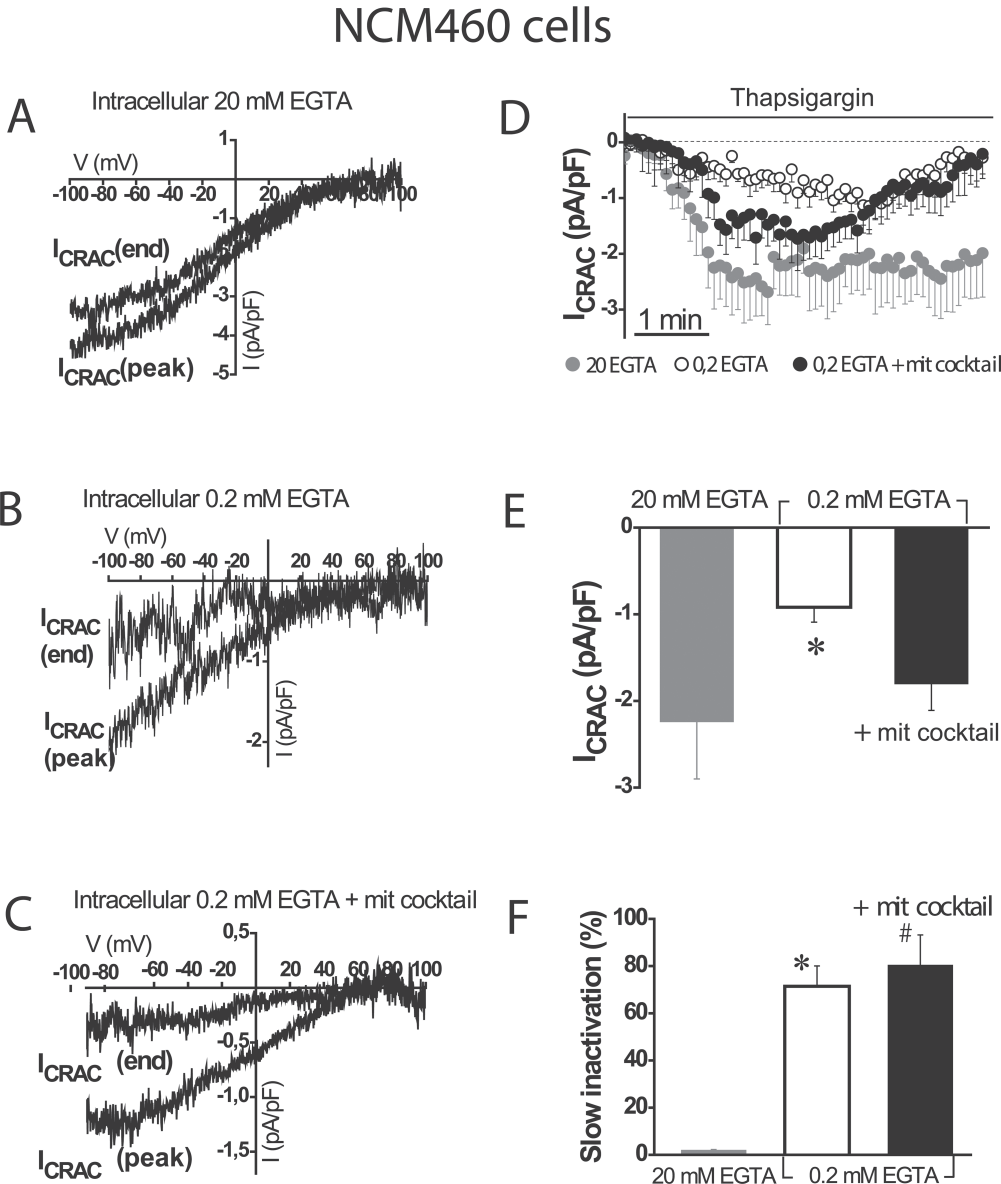
Mitochondria modulate activation of store-operated currents (SOCs) but are not able to prevent the slow, Ca^2+^-dependent inactivation in normal colonic cells I-V relationships of store-operated currents at peak and at the end of the recording period, activated by thapsigargin 1 μM were recorded in NCM460 in intracellular medium containing strong Ca^2+^ buffer (20 mM EGTA) (**A**), physiological Ca^2+^ buffer (0.2 mM EGTA) (**B**) or physiological Ca^2+^ buffer supplemented with a mitochondrial cocktail containing (in mM) 2 pyruvic acid, 2 malic acid, and 1 NaH_2_PO_4_ and intended to maintain efficient mitochondrial respiration (0.2 mM EGTA + mitochondrial cocktail) (**C**, **D**) Average time course recordings of ISOC at –80 mV in NCM460 cells (*n* = 18–24). (**E**) Maximal current amplitude of ISOC in NCM460 (mean ± S.E., *n* = 18–24, *p < 0.05). (**F**) Slow inactivation of current recordings (%) F. *p < 0.05 vs. control; #*p* < 0.5 vs. physiological buffer.

SOCs were activated by depletion of intracellular Ca^2+^ stores with thapsigargin in three different conditions of intracellular Ca^2+^ buffering: (1) strong intracellular Ca^2+^ buffer (EGTA 20 mM) which prevents slow Ca^2+^-dependent inactivation of SOCs, (2) weak Ca^2+^ buffer (EGTA 0.2 mM), and (3) weak Ca^2+^ buffer (EGTA 0.2 mM) supplemented with a mitochondrial cocktail (2 mM pyruvic acid, 2 mM malic acid, and 1 mM NaH_2_PO_4_) previously reported for studying mitochondrial control of SOCs [9]. Although weak Ca^2+^ buffer resembles the physiological buffering, it is necessary supplementing it with the mitochondrial cocktail designed to preserve the full energetic capacity of mitochondria in patch-clamped cells [9]. Figure 2A–2C show representative examples of current/voltage (I/V) relationships of SOCs recorded in the three above mentioned conditions of intracellular Ca^2+^ buffering in normal colonic NCM460 cells. Individual plots display currents obtained from a single cell at maximum amplitude (peak) and at the end of recording period (end). Currents in normal colonic cells were functionally similar to the Ca^2+^-release activated currents (Icrac) reported in other cell types. Currents activated maximally in strong intracellular Ca^2+^ buffer (–2.2 ± 0.7 pA/pF, *n* = 18 cells) and showed no slow inactivation in these conditions (Figure 2D–2F). In weak Ca^2+^ buffer, current maximal amplitude was smaller (–0.9 ± 0.2 pA/pF, *n* = 16 cells) than in strong buffer and showed slow inactivation (Figure 2D). In the weak Ca^2+^ buffer supplemented with mitochondrial cocktail, current amplitude increased (-1.8 ± 0.3 pA/pF, *n* = 24 cells) but showed also slow inactivation (Figure 2D). Average data of current amplitudes and inactivation are shown in Figure 2E and 2F, respectively. The extent of slow inactivation was calculated for each single cell as the percent of current amplitude decrease at the end of recording compared with its maximum value. These results indicate that mitochondria in normal colonic NCM460 cells influence ISOC maximal amplitude but they are unable to prevent the slow Ca^2+^-dependent inactivation even in the presence of the mitochondrial cocktail.

**Figure 3:**
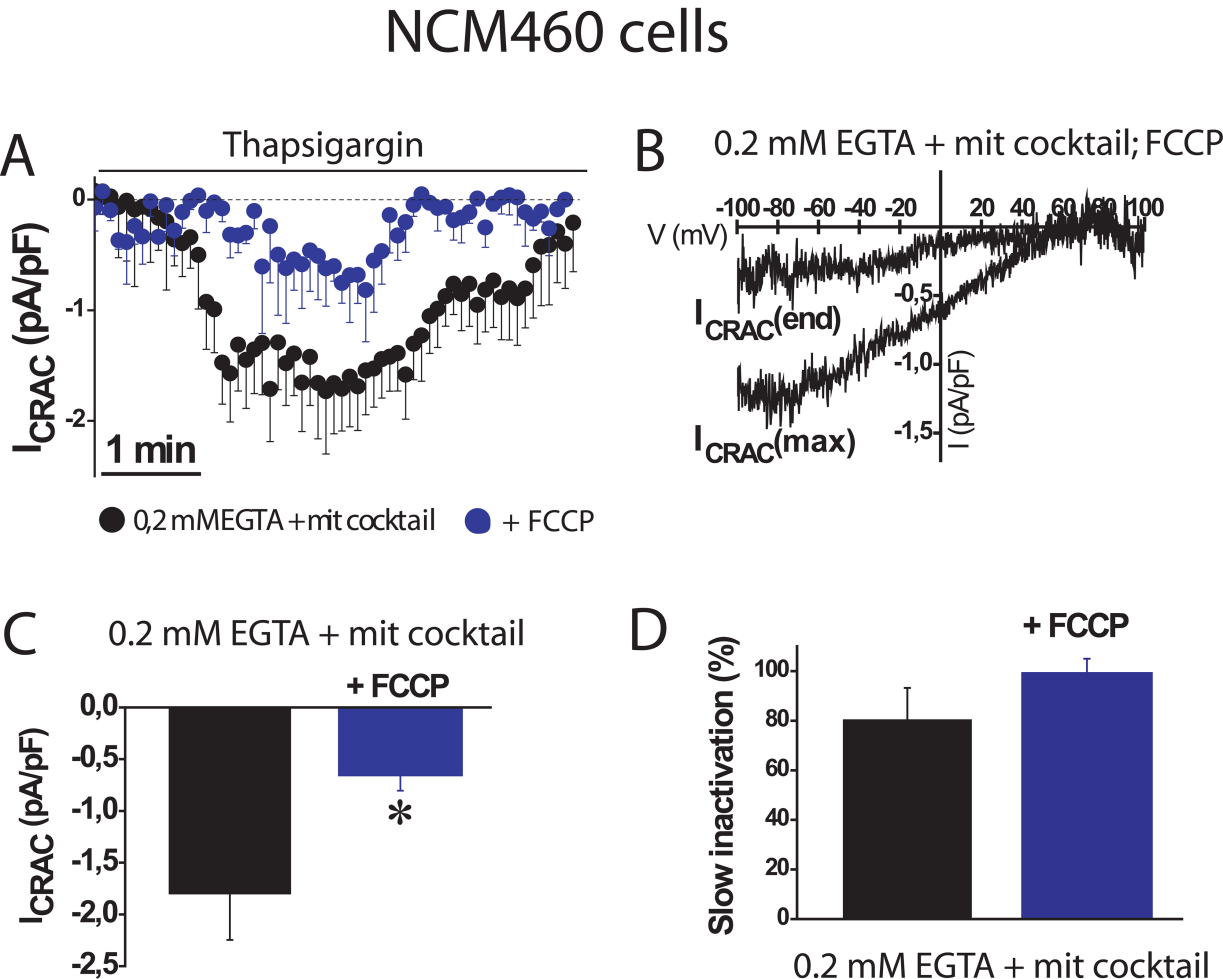
FCCP prevents SOC activation in normal colonic cells ISOC is activated with 1 μM thapsigargin, physiological Ca^2+^ buffer (0.2 mM EGTA) and mitochondrial cocktail (2 pyruvic acid, 2 malic acid, and 1 NaH_2_PO_4_). (**A**, **B**) Average time course recordings of ISOC at -80 mV in NCM460 cells (*n* = 16) treated with 100 nM FCCP. (**C**, **D**) Maximal current amplitude of ISOC in NCM460 (C, mean ± S.E., *n* = 10-24, *p < 0.05). Slow inactivation of these recordings.

To support further the above view, we tested the effects of the mitochondrial uncoupler FCCP on SOC amplitude and inactivation in normal colonic cells. Figure 3 shows that mitochondrial depolarization with FCCP, even in the presence of the mitochondrial cocktail, nearly abolished SOC activity in normal cells (–0.6 ± 0.2 pA/pF, *n* = 10 cells). In addition, slow inactivation of SOCs in normal cells was not prevented by FCCP. These results confirm that mitochondria are essential for current maximal amplitude in normal colonic cells under weak intracellular Ca^2+^ buffering, similar as those found in physiological conditions; however, mitochondria in normal NCM460 cells, either energized or not, are not capable of preventing the slow, Ca^2+-^dependent inactivation of these currents.

### Mitochondria prevents Ca^2+^ dependent inactivation of SOCs in colon cancer cells

**Figure 4:**
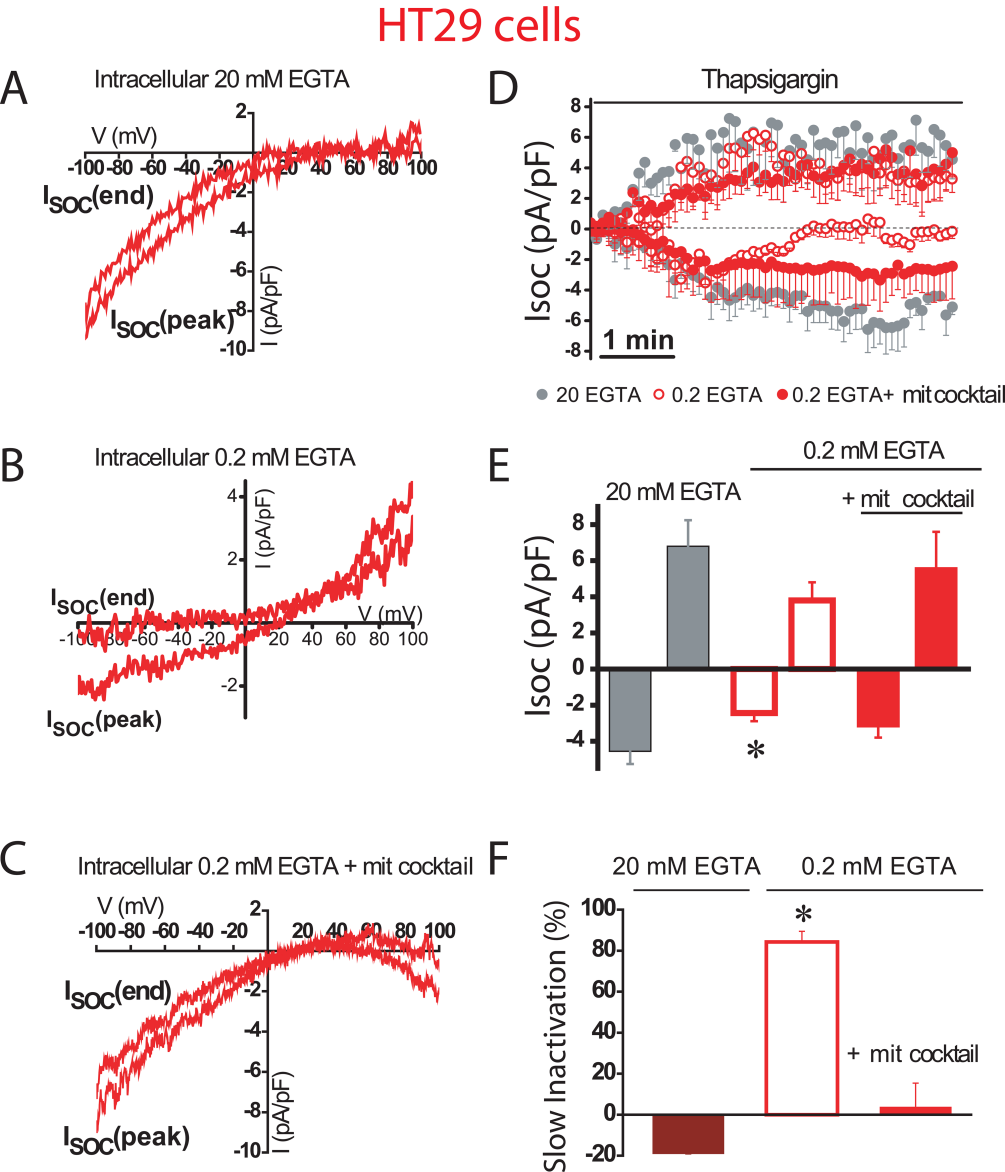
Mitochondria prevents slow, Ca^2+^-dependent inactivation of SOCs in colon cancer cells Store-operated currents activated by thapsigargin 1 μM were recorded in HT29 cells in intracellular medium containing strong Ca^2+^ buffer (20 mM EGTA) (**A**), physiological Ca^2+^ buffer (0.2 mM EGTA) (**B**) or physiological Ca^2+^ buffer supplemented with a mitochondrial cocktail (2 pyruvic acid, 2 malic acid, and 1 NaH_2_PO_4_) intended to maintain efficient mitochondrial respiration (0.2 mM EGTA and mitochondrial cocktail) (**C, D**)Average time course recordings of ISOC at –80 mV in HT29 cells (*n* = 13-31). (**E**) Maximal current amplitude of ISOC (mean ± S.E., *n* = 13–31, *p < 0.05). (**F**) Slow inactivation of these recordings. *p < 0.05.

We next investigated the role of mitochondria in control of SOCs in human colon carcinoma cells. As reported previously [17], SOCs in HT29 colon cancer cells are larger than in NCM460 normal colon cells. We found that in colon cancer HT29 cells, inward SOCs activated maximally (–4.6 ± 0.7 pA/pF and 6.8 ± 1.4 pA/pF, *n* = 31 cells) in strong intracellular Ca^2+^ buffer with no slow inactivation (Figure 4A, 4D). However, in weak intracellular Ca^2+^ buffer, SOCs do not reach the maximal amplitude (–2 ± 0.5 pA/pF, *n* = 15 cells) as the observed in strong Ca^2+^ buffer. In addition, in these conditions of weak Ca^2+^ buffer, SOCs display slow inactivation (Figure 4B, 4D). When weak Ca^2+^ buffer was supplemented with the mitochondrial cocktail, SOC activity increased (–3.2 ± 0.6 pA/pF, *n* = 13 cells) and most importantly, the mitochondria energization prevented the slow inactivation of the inward component of SOCs (Figure 4C, 4D). Interestingly, the outward component of SOCs was not affected by intracellular Ca^2+^ buffering at all. Average data on effects of different buffering conditions on current amplitude and inactivation of SOC are shown in Figure 4E and 4F. Data suggest that, in contrast to normal cell mitochondria, tumor cell mitochondria efficiently prevent the slow, Ca^2+^-dependent inactivation of SOCs. However, mitochondria have a lesser influence on the maximal current amplitude than in normal colonic cells.

**Figure 5:**
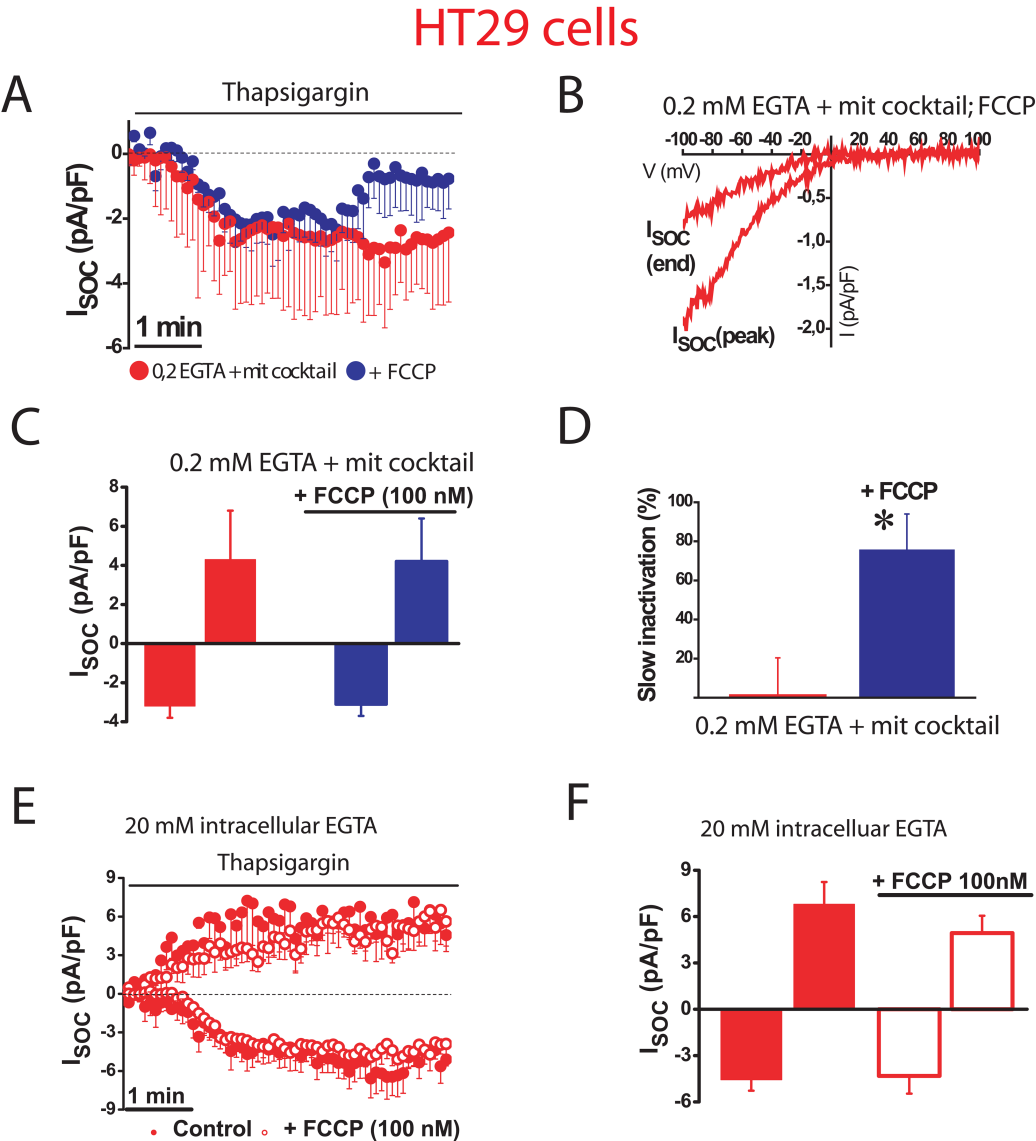
Mitochondrial depolarization promotes SOC inactivation in colon cancer cells ISOC is activated with 1 μM thapsigargin, physiological Ca^2+^ buffer (0.2 mM EGTA) and mitochondrial cocktail (2 pyruvic acid, 2 malic acid, and 1 NaH_2_PO_4_). (**A**, **B**). Average time course recordings of ISOC at –80 mV in HT29 cells (*n* = 13) treated with 100 nM FCCP. (**C**) Maximal current amplitude of ISOC in HT29 cells (mean ± S.E., *n* = 13, *p < 0.05). (**D**) Slow inactivation of these recordings in HT29 cells (*p < 0.05). (**E**) Average time course recordings of ISOC at –80 mV in HT29 cells in strong Ca^2+^ buffer (20 mM EGTA) (*n* = 12) in the absence and the presence of 100 nM FCCP. (**F**) Maximal current amplitude of ISOC in HT29 cells in these conditions (mean ± S.E., *n* = 12, *p < 0.05).

To confirm the role of mitochondria in colon cancer cells, we studied next the effects of FCCP on SOCs using weak Ca^2+^ buffer supplemented with mitochondrial cocktail (Figure 5). FCCP had no significant effect on SOC maximal amplitude (–3.1 ± 0.6 pA/pF, *n* = 13 cells) in tumor cells. However, mitochondria depolarization with FCCP promoted the slow inactivation (Figure 5A–5D) by more than 75% of the maximal current. Consistently, the pro-inactivating effects of FCCP were abolished in the presence of strong intracellular Ca^2+^ buffer (Figure 5E, 5F) indicating that slow inactivation of inward SOCs depends on the ability of tumor cell mitochondria to buffer efficiently Ca^2+^.

### Mitochondrial Ca^2+^ uptake and mitochondrial potential are increased in colon cancer cells relative to normal cells

**Figure 6:**
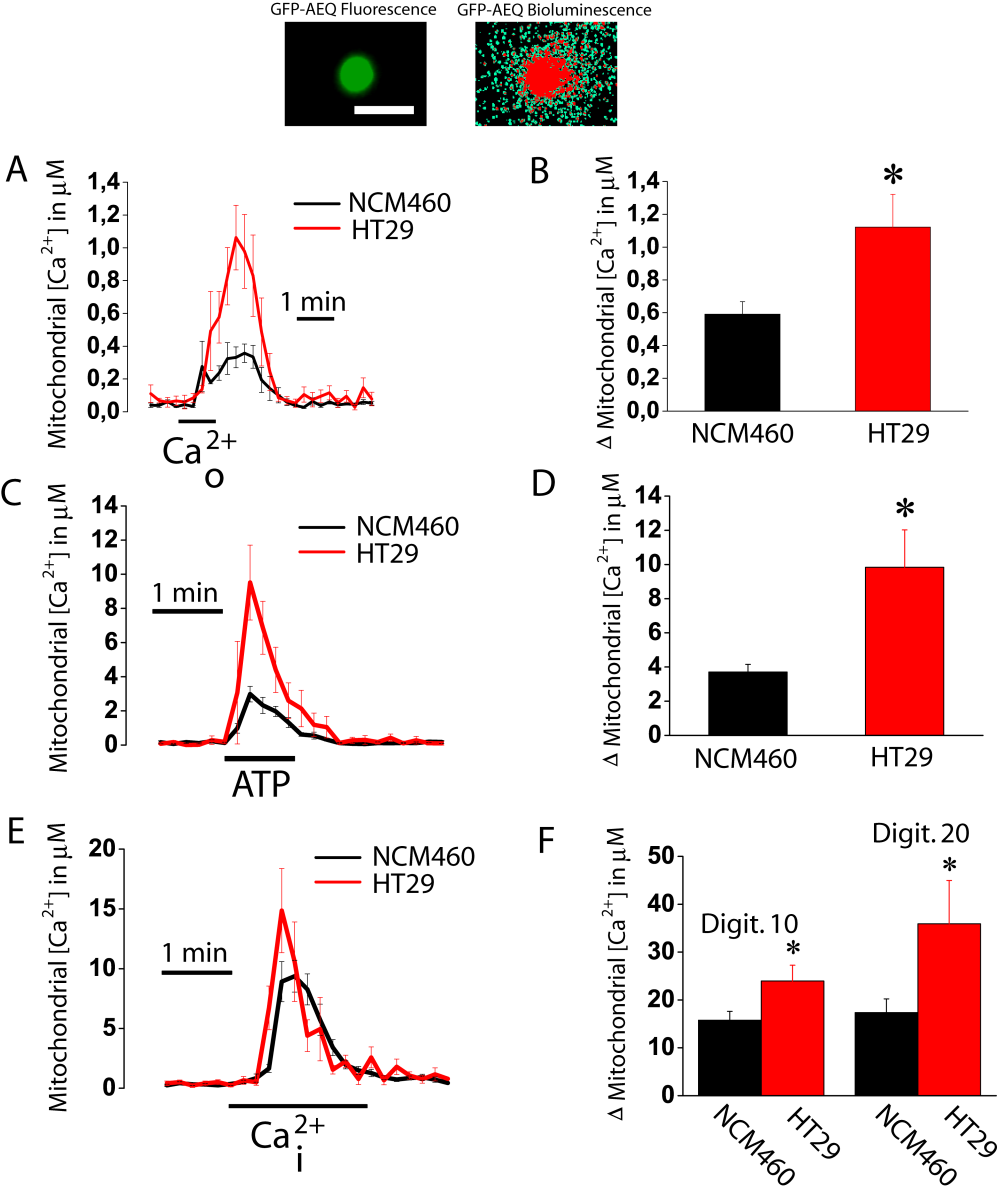
Mitochondrial Ca^2+^ uptake is larger in colon cancer cells than in normal colonic cells Normal (NCM460, black recordings) and colon cancer (HT29 cells, red recording) cells were transfected with GFP-AEQmit and mitochondrial [Ca^2+^] was recorded in individual cells by bioluminescence imaging. Pictures show GFP fluorescence of transfected cells and photonic emissions reflecting mitochondrial [Ca^2+^] (A). Transfected cells were treated with thapsigargin (1 μM, 10 min) in Ca^2+^ free medium to deplete intracellular Ca^2+^ stores. Then cells were subjected to bioluminescence imaging and perfused with Ca^2+^-containing medium (Ca^2+^_o_) to assess the effects of SOCE on mitochondrial Ca^2+^ uptake. Traces and bars are mean ± SEM recordings of mitochondrial [Ca^2+^] values and peak heights, respectively, induced by SOCE (**A, B**). Transfected cells were also stimulated with 100 μM ATP in Ca^2+^ free medium to assess the effects of ATP-induced Ca^2+^ release on mitochondrial Ca^2+^ uptake. Traces and bars are mean ± SEM recordings of mitochondrial [Ca^2+^] values and peak heights, respectively, induced by ATP-induced Ca^2+^ release (**C, D**). Transfected cells were permeabilized in two different conditions, either 10 or 20 μM digitonin in internal medium containing a cytosolic-like Ca^2+^ concentration and then stimulated with the same medium containing 5.7 μM Ca^2+^ mimicking a rise in intracellular Ca^2+^ concentration (Ca^2+^_i_). Traces and bars are mean ± SEM recordings of mitochondrial [Ca^2+^] values and peak heights, respectively, induced by 10 μM Ca^2+^_i_ (**E, F**) of at least 5 experiments. Bars show peak heights of these recordings (mean ± SEM, *n* ≥ 5, *p < 0.05).

We investigated next the ability of mitochondria from normal colonic and colon cancer cells to take up Ca^2+^. For this end, cells were transfected with a plasmid containing a Ca^2+^ probe targeted to mitochondria (mitGA) [25]. Then cells were subjected to bioluminescence imaging to monitor mitochondrial Ca^2+^ concentration ([Ca^2+^]_mit_) after stimulation. Figure 6A shows that extracellular Ca^2+^ added to intact cells previously treated with thapsigargin (to activate SOCE) increases [Ca^2+^]_mit_ in both normal colonic and colon cancer cells. The rise in [Ca^2+^]_mit_ is significantly larger in colon cancer cells than in normal colonic cells (Figure 6A, 6B). This is actually expected as SOCE is much larger in colon cancer cells than in normal cells (Figure 1). However, these data indicate that mitochondria sense Ca^2+^ entering through SOCs in both normal and colon cancer cells. We also tested the effects of physiological stimulation with ATP, an agonist that promotes Ca^2+^ release from intracellular stores, on mitochondrial Ca^2+^ uptake (Figure 6C). Again the rise in mitochondrial Ca^2+^ uptake induced by ATP was larger in cancer cells than in normal colonic cells (Figure 6C, 6D). However, we cannot discard that differences in mitochondrial Ca^2+^ uptake are not due to differences in Ca^2+^ release induced by ATP between normal and cancer cells [17]. To study possible differences in mitochondrial Ca^2+^ uptake we treated cells with different concentrations of digitonin to permeate them and then treat the cells with intracellular medium containing a similar concentration of Ca^2+^ (Figure 6E). We found that intracellular medium containing 5.7 μM Ca^2+^ increased [Ca^2+^]_mit_ in both normal and tumor cells being the rise significantly larger in colon cancer cells than in normal colonic cells (Figure 6E, 6F). These results indicate that colon cancer cell mitochondria take up more Ca^2+^ than normal colonic cell mitochondria. Next we studied possible mechanisms involved in differential mitochondrial Ca^2+^ uptake.

**Figure 7:**
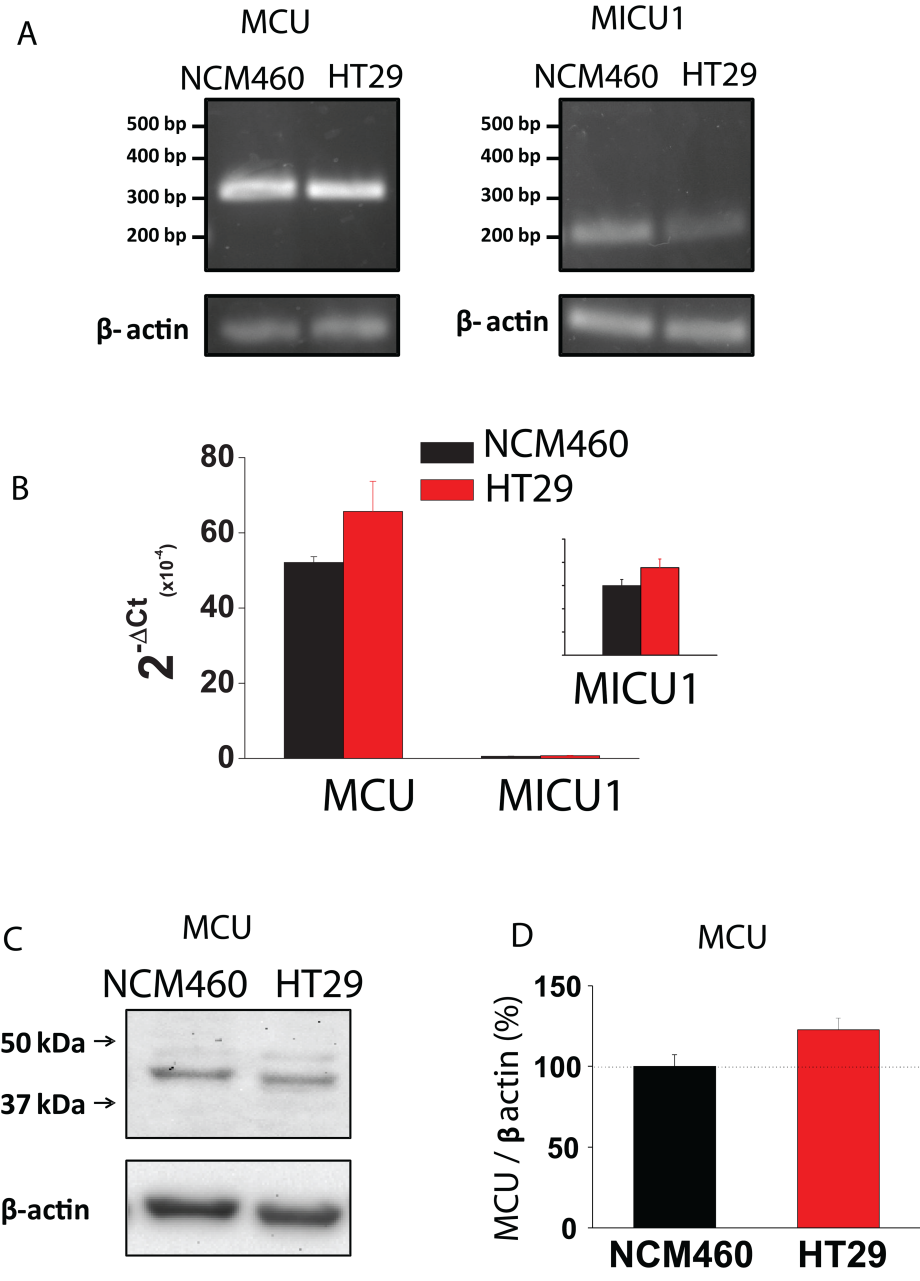
MCU and MICU1 expression levels are similar in NCM460 and HT29 cells (**A, B**) mRNA expression of MCU and MICU1 in normal and tumor cells. Pictures show specific bands of RT-PCR products (A). Bars are mRNA levels normalized to β-actin obtained by qRT-PCR (B, mean ± S.E., *n* ≥ 3, *p < 0,05). (**C, D**) Protein expression of MCU in NCM460 and HT29. Pictures show specific bands of western-blotting products (C). Bars are protein levels normalized to β-actin in HT29 cells relative to NCM460 cells (D, mean ± S.E., *n* = 3, *p < 0.05).

Mitochondrial Ca^2+^ uptake takes place through the mitochondrial Ca^2+^ uniporter (MCU). Accordingly, we studied possible differences in expression of MCU between normal and tumor cells. As shown in Figure 7A, 7B, expression of MCU and its modulatory protein MICU1 are similar in normal and tumor cells at the mRNA level, being mRNA for MCU much more abundant than for MICU1. Therefore, we tested expression of MCU at the protein level using western blotting. Figure 7C, 7D shows that MCU protein expression is similar in normal and tumor cells. Thus, it is unlikely that differences in mitochondrial Ca^2+^ uptake between normal and colon cancer cells are due to differences in expression of MCU and/or its modulatory regulator MICU1.

**Figure 8:**
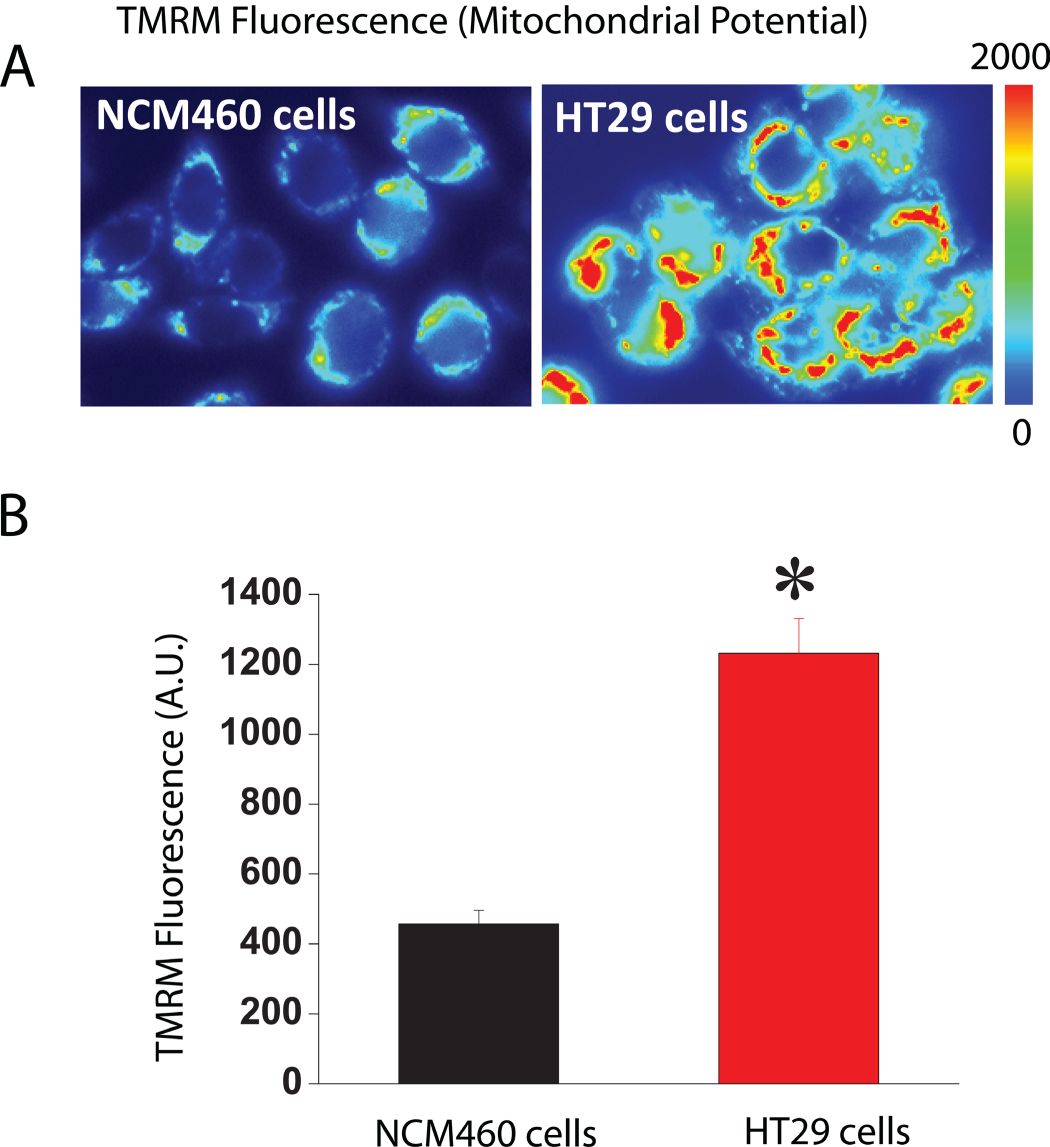
Mitochondrial potential (ΔΨ) is larger in colon cancer cells than in normal colonic cells Normal and colon cancer cells were incubated with the mitochondrial potential probe TMRM (50 nM, 15 min) before fluorescence imaging using the rhodamine filters. (**A**) Pictures reflecting mitochondrial potential coded in pseudocolor. (**B**) Bars show mean ± SEM of 6 independent experiments (*p < 0.05). Black bar, normal cells. Red bar, colon cancer cells.

The other critical determinant of mitochondrial Ca^2+^ uptake in addition to MCU is ΔΨ, the driving force for Ca^2+^ transport from cytosol to mitochondria, two compartments with similar Ca^2+^ concentration at rest (about 100 nM). We asked therefore for possible differences in ΔΨ between normal and colon cancer cells. For this end, we used fluorescence imaging of cells treated with TMRM, a fluorescence probe that accumulates inside mitochondria according to ΔΨ [20, 26]. As shown in Figure 8, colon cancer cells accumulate much more TMRM than normal cells consistently with enhanced ΔΨ in tumor cells. These results are consistent with previous observations in most tumor cells that display increased ΔΨ associated to the Warburg effect [27]. Thus, when taken together, our data suggest that the larger mitochondrial Ca^2+^ uptake of colon cancer cells relative to normal cells is likely due to enhanced ΔΨ associated to the Warburg effect.

### Selected NSAIDs depolarize mitochondria and inhibit mitochondrial Ca^2+^ uptake in colon cancer cells

**Figure 9:**
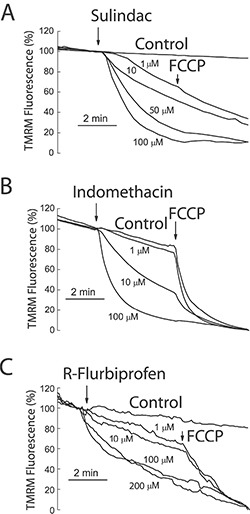
NSAIDs depolarize mitochondria in colon cancer cells HT29 colon cancer cells were incubated with the mitochondrial potential probe TMRM (50 nM, 15 min) before fluorescence imaging using the rhodamine filters. Cells were continuously perfused with standard external medium. Cells were treated with medium containing solvent (Control) or different concentrations of sulindac sulphide (**A**), indomethacin (**B**) and R-flurbiprofen (**C**). In some cases, FCCP 10 μM was added to collapse ΔΨ. Data are average recordings normalized to the value before addition of treatments and representative of 3 independent experiments for each treatment.

**Figure 10:**
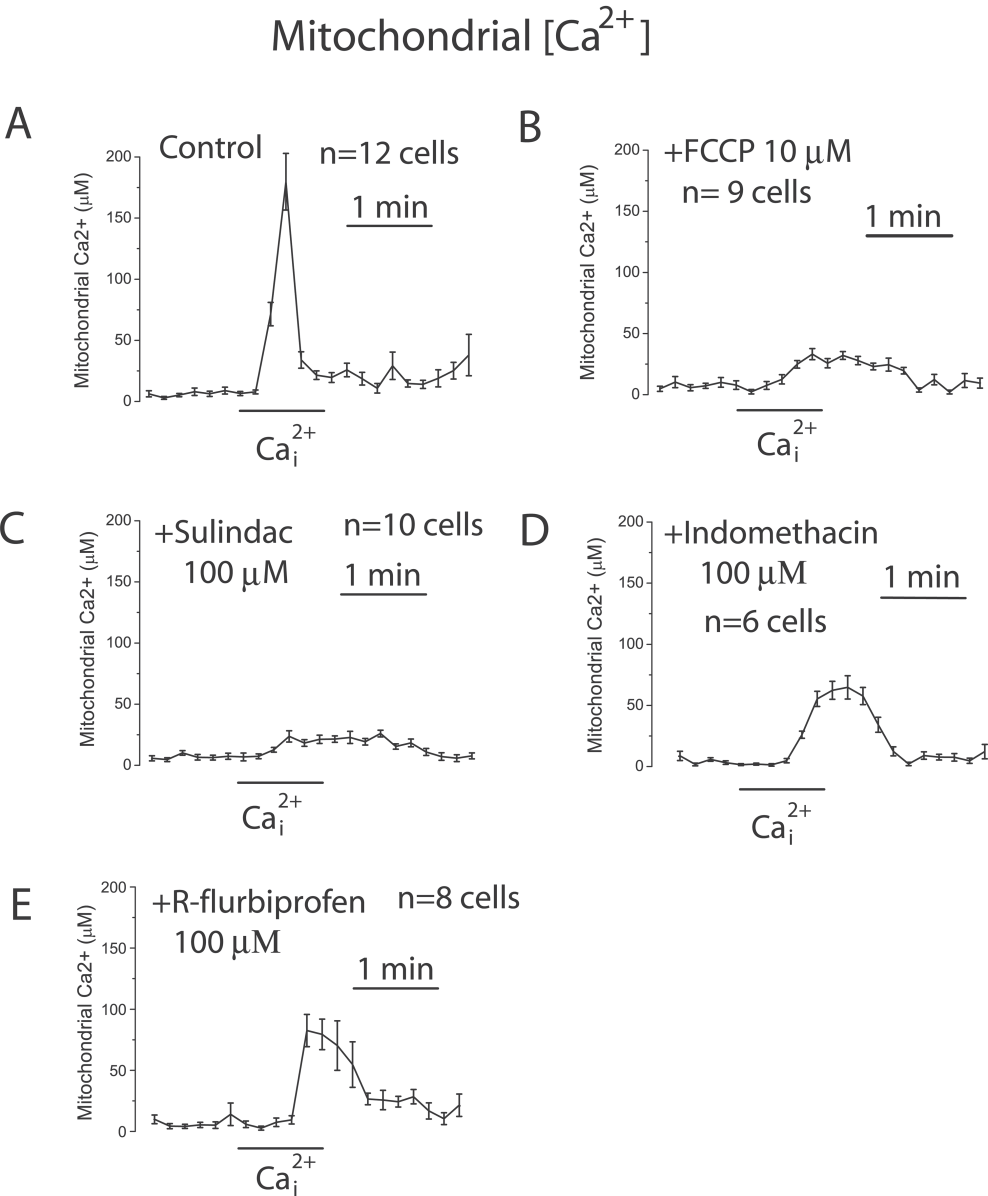
NSAIDs inhibit mitochondrial Ca^2+^ uptake in colon cancer cells HT29 cells were transfected with mutated GFP-AEQ targeted to mitochondria. Next day cells were incubated with celenterazine and then subjected to bioluminescence imaging for monitoring mitochondrial [Ca^2+^]. Cells were incubated in digitonin in intracellular medium containing 200 nM [Ca^2+^] and then stimulated with 5.7 μM to induce mitochondrial Ca^2+^ uptake in medium containing solvent (**A**), 100 nM FCCP (**B**), or 100 μM sulindac sulphide (**C**), or 100 μM (Indomethacin) (**D**), or 100 μM (R-flurbiprofen) (**E**). Traces are mean ± SEM recordings of mitochondrial [Ca^2+^] induced of transfected cells identified by the GFP fluorescence corresponding to 8-12 individual cells recorded in 3 to 5 independent experiments for each treatment.

To support further the above view, we targeted ΔΨ using a series of non-steroidal anti-inflammatory drugs (NSAIDs) that, acting as mild mitochondria uncouplers [28], may depolarize mitochondria and hence modulate mitochondrial Ca^2+^ uptake, SOCs and SOCE in colon cancer cells. Figure 9 shows that classic NSAIDs sulindac sulphide and indomethacin, as well as R-flurbiprofen, an enantiomer of flurbiprofen lacking anti-inflammatory activity, decreased TMRM fluorescence in colon cancer cells in a dose-dependent manner, consistently with mitochondria depolarization. These results are consistent with previous observations of mitochondrial depolarization induced by these compounds in other cells types [20–22] including neurons [29–31]. Furthermore, bioluminescence recordings of [Ca^2+^]_mit_ in colon cancer cells treated with digitonin and intracellular medium containing 5.7 μM Ca^2+^ revealed that the same NSAIDs inhibit mitochondrial Ca^2+^ uptake at the same concentrations that depolarize mitochondria and to a similar extent than low concentrations of FCCP (Figure 10). These results are consistent with the important role of ΔΨ in mitochondrial Ca^2+^ uptake reported previously [20]. As NSAIDs counteracted the effects of the Warburg effect on ΔΨ we asked next for the effects of these compounds on SOCs.

### Selected NSAIDs promote SOC inactivation and inhibit SOCE and cell proliferation in normal and colon cancer cells

**Figure 11:**
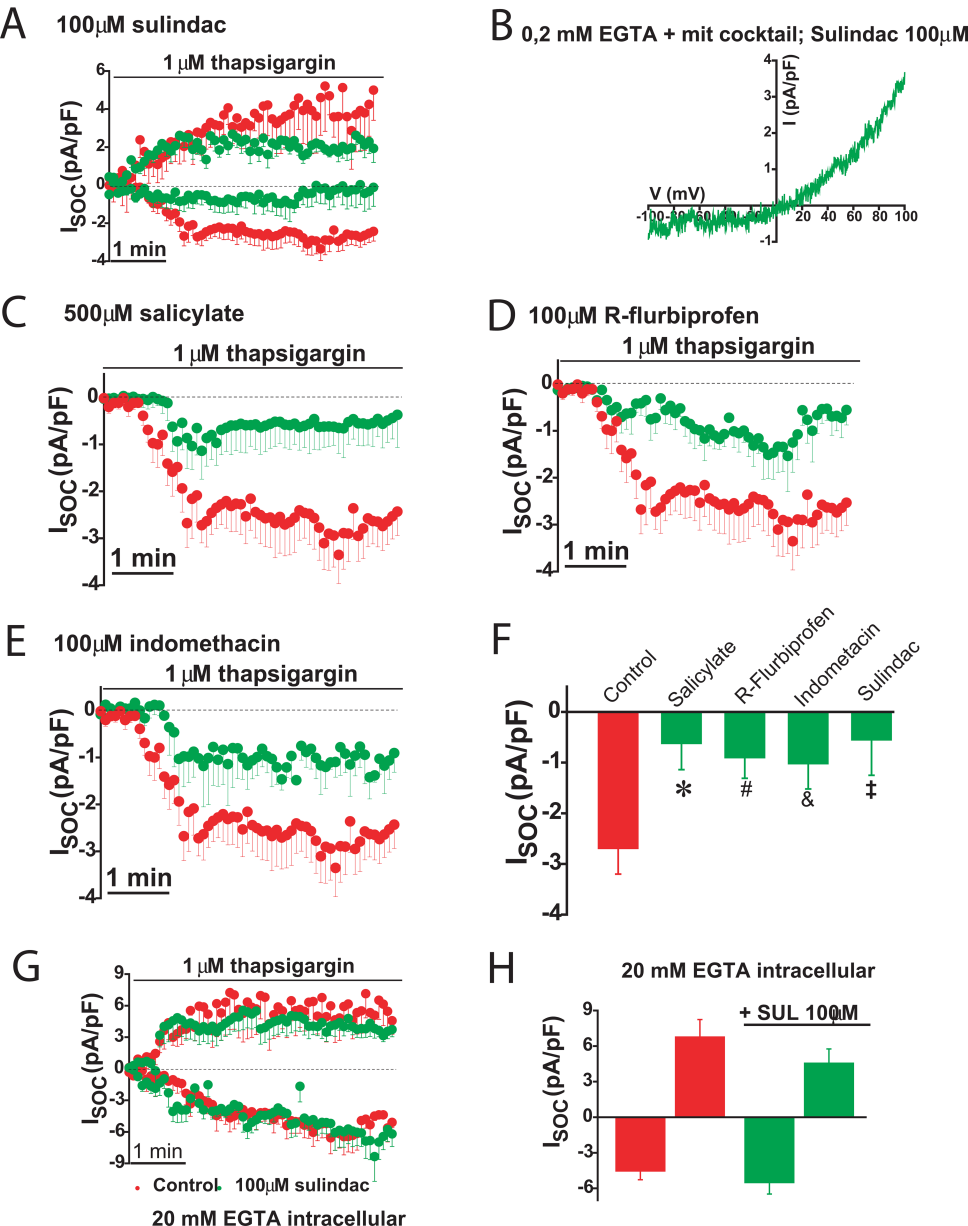
NSAIDs and FCCP promotes slow dependent inactivation in colon cancer cells Thapsigargin activated SOCs were recorded in HT29 cells in physiological buffer (0.2 mM EGTA + mitochondrial cocktail) and the presence of either solvent (red recordings) or in the presence of 100 μM sulindac sulphide (green recordings, (**A**). I-V relationship of a SOC recorded in a representative cell showing inward and outward component in the presence of sulindac (**B**). SOCs were also recorded in HT29 cells in the presence of solvent (red recordings) or different NSAIDs (green recordings) including 500 μM salicylate (**C**), 100 μM R-flurbiprofen (**D**) or 100 μM indomethacin (**E**). Data are mean ± SEM values of current density (pA/pF) recorded in 10 to 14 cells. (**F**) shows mean ± SEM values or peak current in control cells and cells treated with the different NSAIDs. *p < 0.05. (**G**) shows currents recorded in high intracellular Ca^2+^ buffer (20 mM EGTA) in the presence of solvent (red recording) or 100 μM sulindac sulphide (green recording). Data are mean ± SEM values of 14 different cells. (**H**) shows current density values for inward and outward components of control cells and cells treated with sulindac sulphide.

The effects of selected NSAIDs on SOCs were investigated in colon cancer cells using patch-clamp electrophysiology. We found that the aspirin metabolite salicylate, sulindac, indomethacin and R-flurbiprofen decreased SOCs amplitude and/or promoted SOCs inactivation in colon cancer cells (Figure 11A–11F) consistently with the effects of these compounds on ΔΨ and mitochondrial Ca^2+^ uptake [20]. Furthermore, the effects of NSAIDs on SOCs were removed by increasing the intracellular Ca^2+^ buffering capacity. These results indicate that NSAIDs do not inhibit SOCs directly but instead they promote their slow, Ca^2+^-dependent inactivation.

**Figure 12:**
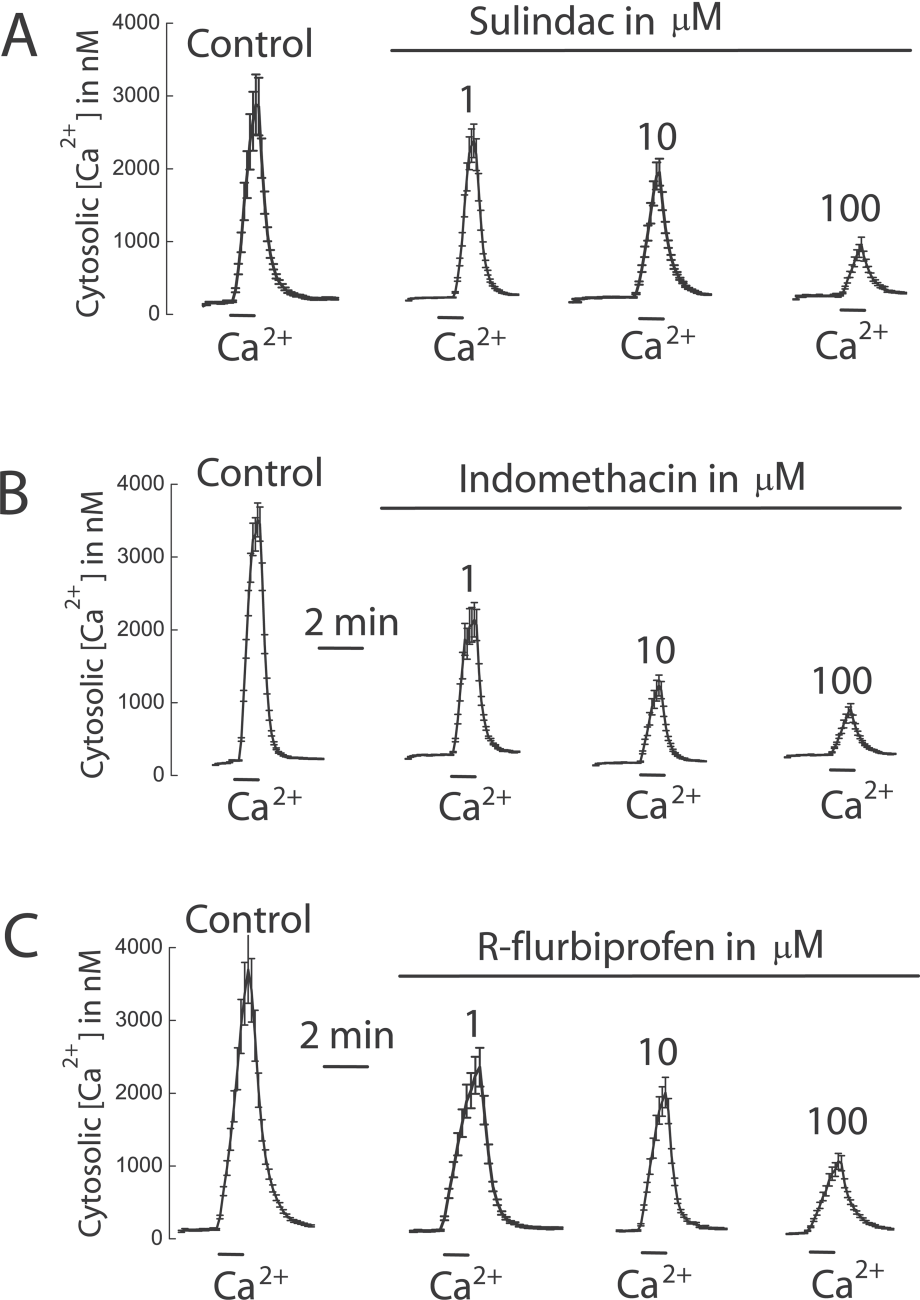
NSAIDs inhibit SOCE in colon cancer cells HT29 cells were incubated with Fura4F/AM (4 μM, 60 min) at room temperature. Then cells were treated with thapsigargin (1 μN, 10 min) in Ca^2+^ free medium and then subjected to fluorescence imaging for monitoring of cytosolic [Ca^2+^]. Addition of 1 mM Ca^2+^ induce large increases in [Ca^2+^]_cyt_ corresponding to SOCE. Cells were treated with solvent or different concentrations of sulindac sulphide (**A**), indomethacin (**B**) or R-flurbiprofen (**C**). Recordings are representative mean ± SEM values of [Ca^2+^]_cyt_ of different cell batches treated with solvent or different concentrations of the above mentioned compounds. Data are representative of at least three independent experiments with each compound.

**Figure 13:**
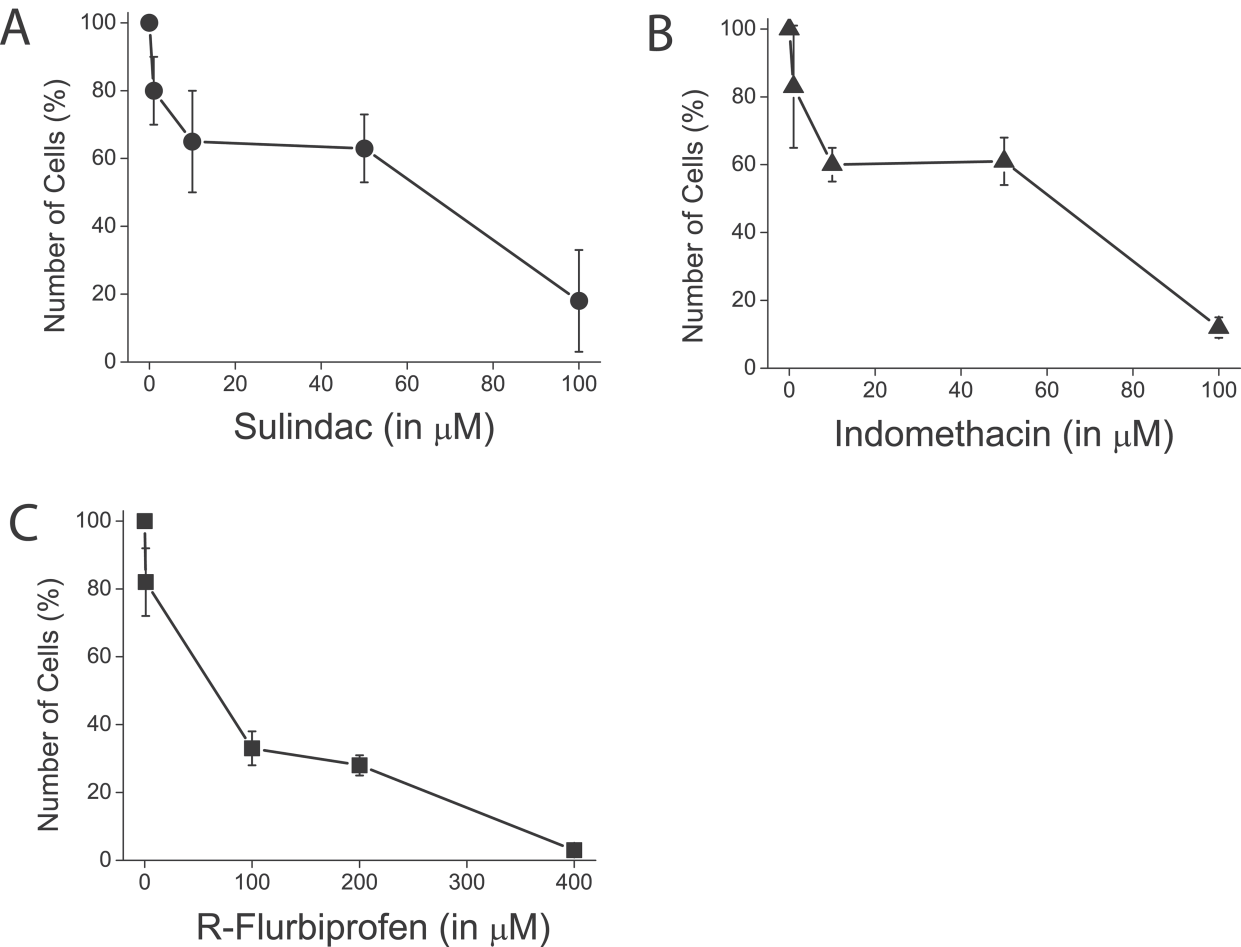
NSAIDs inhibit colon cancer cell proliferation HT29 cells were plated in culture medium containing solvent of different concentrations of sulindac sulphide (**A**), indomethacin (**B**) and R-flurobiprofen (**C**) and the number of cells was estimated at plating and after 96 h. The number of cells at the end of the 96 h period is shown as a percent value (mean ± SEM) of the number of cells at plating. *p < 0.05. Percent of dead cells was lower than 5 % in all cases as shown by trypan blue staining.

We tested also the effects of selected NSAIDs on SOCE and cell proliferation in colon cancer cells and normal cells. Figure 12 shows that NSAIDs inhibit SOCE in colon cancer cells in a dose dependent manner. Again these effects were reproduced by R-flurbiprofen lacking anti-inflammatory activity but showing the same mitochondria depolarizing activity. The same compounds inhibited cell proliferation in colon cancer cells in a dose-dependent manner (Figure 13) consistently with the role of SOCE in cell proliferation in colon cancer cells [17].

**Figure 14:**
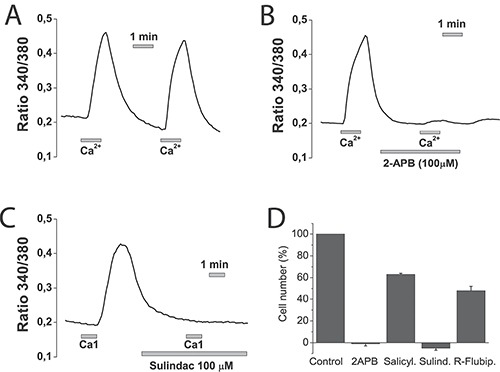
NSAIDs inhibit SOCE and cell proliferation in normal NCM460 cells NCM460 cells were loaded with fura2/AM and treated with thapsigargin for depleting intracellular Ca^2+^. Then cells were subjected to Ca^2+^ imaging for monitoring of SOCE. Cells were perfused in Ca^2+^-free medium and then exposed to two consecutive, 1 min pulses of medium containing 1 mM Ca^2+^. To test the effects of selected compounds cells, before the second 1 mM calcium pulses, cells were treated with solvent (**A**), 100 μM 2APB (**B**) or 100 μM sulindac sulphide (**C**). Data are representative recordings of at least three independent recording. (**D**) NCM460 cells were plated in culture medium containing solvent or 2-APB (100 μM), salicylate (500 μM), sulindac sulphide (100 μM) or R-flurbiprofen (100 μM) and the number of cells was estimated at plating and after 96 h. The number of cells at the end of the 96 h period is shown as a percent value (mean ± SEM) of the number of cells at plating. *p < 0.05. Percent of dead cells was lower than 10% in all cases except for sulindac sulphide that amounted 14 ± 2 % as shown by trypan blue staining.

As in normal colonic cells SOCE and SOCs are also modulated by mitochondria and involved in cell proliferation, we tested also the NSAIDs on SOCE and cell proliferation in these cells. In NCM460 cells, SOCE is nearly abolished by SOCE antagonist 2-APB and sulindac sulphide, the stronger mitochondrial uncoupler (Figure 14). Consistently, NCM460 cell proliferation was largely prevented by both 2-APB and sulindac sulphide. Other NSAIDs like salicylate and R-flurbiprofen inhibited cell proliferation to a lower extent consistently with their lower depolarizing capability (Figure 14).

## DISCUSSION

We have reported recently that colon cancer cells show a remarkable remodeling of intracellular Ca^2+^ homeostasis that contributes to cancer hallmarks including enhanced cell proliferation, cell invasion and resistance to cell death. Most salient features of remodeling are increased SOCE and enhanced and modified SOCs along the partial depletion of intracellular Ca^2+^ stores [17]. Another signature of most tumor cells is the Warburg effect by which affected tumor cells rely mostly on anaerobic glycolysis for energy production because of inhibition of oxidative phosphorylation [1, 2]. However, although mitochondrial ATP synthesis is prevented in most tumor cells, mitochondria actually display enhanced ΔΨ [27]. Mechanisms for enhanced ΔΨ are not completely understood but lack or inhibition of the H^+^-ATP synthase that dissipates ΔΨ during aerobic ATP synthesis probably plays a pivotal role in this regard [3]. Regardless of the mechanism, as ΔΨ constitutes the driving force for mitochondrial Ca^2+^ uptake and this process has been involved in control of SOCE, it is possible that colon cancer mitochondria modulate SOCE in a different way than normal cell mitochondria. We show that, consistently with enhanced ΔΨ of tumor cells, mitochondria from colon cancer cells take up more Ca^2+^ than mitochondria of normal cells and efficiently prevent the slow, Ca^2+^-dependent inactivation of SOCs, thus enhancing and sustaining SOCE in colon cancer cells. Mitochondria are also relevant for SOCs in normal cells but in a different fashion. In normal cells, mitochondria are not able to prevent the slow, Ca^2+^-dependent inactivation of CRAC channels and currents inactivate regardless of mitochondrial status. Therefore, mitochondria modulate differentially SOCs in normal and colon cancer cells. Nevertheless, we must acknowledge that these results have been obtained in two cell lines representing normal and colon cancer cells and they should be confirmed also in normal and colon cancer cells obtained for patients.

Mitochondrial Ca^2+^ uptake depends on multiple factors including 1) Ca^2+^ transport by the MCU and regulatory proteins, 2) ΔΨ, the driving force for mitochondrial Ca^2+^ uptake and 3) the relative proximity and/or coupling between mitochondria and Ca^2+^ channels in the plasma membrane and/or the ER. Our results indicate that MCU levels are similar in normal and colon cancer cells. However, as consequence of the Warburg effect, tumor cells display a much larger driving force for mitochondrial Ca^2+^ uptake that may contribute to explain enhanced mitochondrial Ca^2+^ uptake observed in tumor cells. In addition to ΔΨ, differential sensing by mitochondria of Ca^2+^ microdomains formed near SOC channels may also contribute to differences in mitochondrial Ca^2+^ uptake and hence, SOCE. In this regard, the larger SOCE observed in tumor cells and increased expression of associated molecular players as Orai1, TRPC1 and Stim1, likely allows formation of larger Ca^2+^ domains near mitochondria. Inasmuch as the MCU is a Ca^2+^-activated, Ca^2+^ channel [6, 7], high Ca^2+^ domains in tumor cells should lead to more efficient mitochondrial Ca^2+^ uptake [32] followed by prevention of the ensuing slow, Ca^2+^-dependent inactivation of SOCs. In normal colonic cells, however, the lower expression of Stim1 and Orai1 result in diminished SOCE, less capable of promoting high Ca^2+^ domains, thus limiting mitochondrial Ca^2+^ uptake, particularly in the face of a decreased ΔΨ of normal cell´s mitochondria. Notice in this regard that, if a very high intracellular Ca^2+^ buffer is used for recording of SOCs in normal cells, currents do not inactivate suggesting that SOC inactivation in normal cells is because of lack of enough Ca^2+^ buffering capacity by surrounding mitochondria rather than channels being intrinsically inactivating. However, mitochondrial Ca^2+^ uptake is required for maximal SOC activity in normal cells since, in physiological buffer, currents are extremely small and inactivating. Therefore, in normal cells, mitochondria are necessary for SOC activity but currents inactivate. In contrast, in tumor cells, mitochondria are able to prevent the slow, Ca^2+^-dependent inactivation of SOCs. This effect may help to sustain increased SOCE over time. In fact, increased expression of molecular players involved in SOCE, particularly Orai1 and Stim1, may not be enough to sustain Ca^2+^ in colon cancer cells. Overexpression of these channels may enlarge Ca^2+^ entry but this favors inactivation and, unless intracellular buffer capacity increases along channel expression, the Ca^2+^-dependent inactivation would short cut the entry of Ca^2+^. Our results suggest that this extra buffering capacity may be provided by the enhanced ΔΨ linked to the Warburg effect, the metabolic signature of most tumor cells.

Mitochondrial location relative to the plasma membrane (where SOCs are) and ER (where the gating mechanism for SOC opening is started) might influence SOC activation and inactivation differentially in normal and colon cancer cells. For example, mitochondria located close to ER could exert local modulation of SOCE [33]. Mitochondrial Ca^2+^ buffering at their interface with ER might decrease Ca^2+^ refill of stores, thus sustaining SOCE for longer periods of time [34]. However, there is evidence about opposite effect, i.e. mitochondria support stores refilling by releasing Ca^2+^ close to SERCA pumps in the ER [35, 36]. Although paradoxical, it has been proposed that mitochondrial may play both roles in different temporal windows. Whereas mitochondrial Ca^2+^ buffering modulates activation and sustains SOCE, mitochondrial release of Ca^2+^ close to SERCA sites promotes refill of stores inactivating SOCE [37].

Even more relevant may be mitochondria close to plasma membrane that can take up Ca^2+^ entering through plasma membrane Ca^2+^ channels. This has been studied in detail in T cells where activation of SOCE leads to redistribution of mitochondria towards plasma membrane [38]. Moreover, in these cells, mitochondria display preference to translocate close to immunological synapses, and allow higher Ca^2+^ influx in specific microdomains. Interestingly, those regions rich in mitochondria have higher Ca^2+^ influx [39]. If this is a general mechanism, it will be expected that linking mitochondria to plasma membrane could generate higher SOCE in the whole cell. Surprisingly, in endothelial cells, the artificial redistribution of mitochondria towards the plasma membrane decreased SOCE [40]. Furthermore, in HeLa cells, close contact of mitochondria to Ca^2+^ influx channels is not required for SOCE modulation [41]. These data indicate that although in immune cells localization of mitochondria is crucial to modulate SOCE, this may not apply similarly to other cell types. Interestingly, the adenomatous polyposis coli (APC), a protein responsible for familial adenomatous polyposis that plays also a rate-limiting role in the majority of sporadic colorectal cancers, is a mitochondrial protein that binds the Miro/Milton motor complex involved in mitochondria transport to the plasma membrane [42]. Thus, APC deficits linked to colon cancer may influence mitochondrial control of SOCE. Further research is required to address whether mitochondria localization and/or coupling to ER and/or plasma membrane may influence differentially SOCE in normal and colon cancer cells

A novel mechanism of mitochondria control of SOCE has been recently revealed mediated by the mitochondrial Na^+^/Ca^2+^ exchanger NCLX [43]. According to this model, store-associated activation of Na^+^ influx across the plasma membrane is essential for activation of the mitochondrial Na^+^/Ca^2+^ exchanger (NCLX) that enables Ca^2+^ extrusion from mitochondria. In the absence of Na^+^ influx, lack of mitochondrial Ca^2+^ clearance promotes reactive oxygen species (ROS) and oxidation of cysteine C195 in Orai1, leading to SOC inactivation. Interestingly, SOCs in colon cancer cells, but not in normal cells, involve Na^+^ currents mediated by TRPC1 that form a channel complex with Orai1 [17, 44], thus allowing Na^+^ influx. Accordingly, it is temptying to speculate whether this redox mechanism mediated by NCLX may contribute to sustain SOCE in colon cancer cells, but not in normal cells. Further research is required to support this possibility.

The important role of mitochondria in sustaining SOCs and SOCE in colon cancer cells may have important therapeutic implications. In this scenario, we can expect that targeting enhanced ΔΨ could reverse the sustaining effects of tumor mitochondria, thus leading to transient SOCs and decreased SOCE. We show here that this is indeed the case for several NSAIDs that, working as mild mitochondrial uncouplers, depolarize partially mitochondria and inhibit mitochondrial Ca^2+^ uptake, thus leading to slow SOC inactivation and inhibition of SOCE and cell proliferation. Consistently, the slow inactivation achieved by these compounds is only observed in weak Ca^2+^ buffer but not in strong Ca^2+^ buffer, a condition that prevents the slow, Ca^2+^-dependent inactivation of SOCs regardless of mitochondrial functional status. Therefore, the above data exclude the possibility that NSAIDs acted by inhibiting channels directly and indicate that they, instead, NSAIDs favor the Ca^2+^-dependent inactivation in a mitochondria-dependent fashion.

Overwhelming evidence indicate that aspirin and other NSAIDs may protect against colon cancer and some of these compounds are actually under consideration for colon cancer chemoprevention in high risk individuals by the U.S. Preventive Services Task Force [23]. A few years ago, we suggested that salicylate, the major aspirin metabolite, could prevent mitochondrial Ca^2+^ uptake and inhibit SOCE in a similar way [19, 20]. Now we provide direct electrophysiological evidence that salicylate and other NSAIDs, including sulindac and indomethacin, promote the slow Ca^2+^-dependent inactivation of SOCs in colon cancer cells. These effects are not related to the anti-inflammatory properties of these compounds as they are also mimicked by structural analogues like R-flurbiprofen lacking anti-inflammatory activity. In addition, these effects depend on the ability of NSAIDs to prevent mitochondrial Ca^2+^ uptake working as mild mitochondrial uncouplers [28]. Consistently, effects of NSAIDs are abolished in strong Ca^2+^ buffer where SOCs do not inactivate regardless of the functional mitochondrial status. Our results do not exclude that aspirin and other NSAIDs may prevent cancer acting on additional targets as, for instance, inhibition of COX2 in selected tumors.

In summary, we conclude that mitochondria and changes in ΔΨ related to the Warburg effect, the metabolic signature of most tumor cells, prevent the slow, Ca^2+^-dependent inactivation of store-operated currents in colon cancer cells but not in normal cells, thus sustaining and enhancing store-operated Ca^2+^ entry and related cancer hallmarks. These effects are largely counteracted by selected NSAIDs that promote SOC inactivation in a mitochondria-dependent manner. Thus, the Warburg effect may contribute to tumor cell growth by promoting Ca^2+^ remodeling in colon cancer.

## MATERIALS AND METHODS

### Materials

HT29 cells were donated by Dr. J.C. Fernández-Checa (CSIC, Barcelona, Spain). NCM460 cells were obtained from INCELL Corporation (San Antonio, TX, USA). Dulbecco's Modified Eagle's Medium (DMEM), Penicillin, streptomycin, L-glutamine and fetal bovine serum were from Lonza (Basel, Switzerland). M3:10^TM^ medium was from INCELL Corporation (San Antonio, TX, USA). Detachin was from Gelantis (San Diego, CA, USA). Fura2/AM and Fura4F/AM were from Invitrogen (Carlsbad, CA, USA). AINEs and 2-APB are from Sigma-Aldrich (Madrid, Spain). 2-APThapsigargin is from Alomone Labs (Jerusalem, Israel). Anti β-actin was from ABcam (Cambridge, UK) anti MCU is from Santa Cruz Biotechnology (Dallas, TX, USA). SYBR green I was from Kappa Biosystems (Boston, MA, USA). Primers were obtained from Thermo Scientific (Ulm, Germany). mitGA plasmid was kindly donated by P. Brület (CNRS, France).

### Cell culture

Cells were cultured in DMEM 1 g/L glucose or in M3:10^TM^ media as reported previously [15]. Culture media were supplemented with 1% Penicillin-Strepromycin, 1% L-glutamine and 10% fetal bovine serum. Cells were maintained in standard conditions (37°C, 10% CO_2_) and subcultured once a week. All cells were used at passages 3 to 10.

### Cytosolic Ca^2+^ imaging

[Ca^2+^]_cyt_ was monitored as reported previously [15] by fluorescence imaging of cells using an inverted Zeiss Axiovert microscope equipped with a OrcaER Hamamatsu digital camera (Hamamatsu Photonics France). Cells were loaded with Fura2/AM or Fura4F/AM (4 μM, 60 min) in saline external medium (SEM) containing (in mM): 145 NaCl, 5 KCl, 1 CaCl_2_, 1 MgCl_2_, glucose 10, Hepes/Na^+^ 10 (pH 7.42). Cells were continuously perfused with SEM at 37°C and epi-illuminated alternately at 340 and 380 nm using band pass filters located in a filter wheel. Light emitted above 520 nm at both excitation lights was filtered by a dichroic mirror and collected every 5–10 s with a 40x, 1.4 NA, oil objective. For SOCE, previously to Ca^2+^ imaging, cells were washed twice and treated with thapsigargin (1 μM, 10 min) in SEM without Ca^2+^ and containing 0.5 mM EGTA. Then, cells were placed in the stage of an inverted microscope and perfused with SEM without Ca^2+^. To activate SOCE, cells were exposed to SEM containing 1 mM Ca^2+^. For treatment, cells were perfused with NSAIDs and FCCP in Ca^2+^-free SEM for 5 min and then perfusion was switched to SEM containing NSAID and Ca^2+^. Fura2 was used to compare effects of FCCP on SOCE in normal and colon cancer cells. However, to assess the effects of NSAIDs on the large SOCE observed in colon cancer cells, Fura4F, a dye with lower affinity for Ca^2+^, was used instead.

### Cell proliferation

Cells were plated in 6 well plates at a density of 10 × 10^5^ cells and incubated with supplemented DMEM or the same medium contained test substances. Cells in wells were counted in triplicate at time 0 h and after 72, or 96 h using a hemocytometer. Cell viability was estimated using trypan blue staining.

### Electrophysiological recordings

SOCs were recorded using a Port-a-patch planar patch-clamp system (Nanion Technologies, Munich, Germany) in the whole-cell, voltage-clamp configuration, at room temperature (20 ± 2°C) as reported previously [15]. Cultured cells (3–5 days after plating) were detached with Detachin and suspended at a cell density of 1–5 × 10^6^ cells/mL in external recording solution containing (in mM): 145 NaCl, 2.8 KCl, 2 MgCl_2_, 10 CaCl_2_, 10 HEPES, 10 D-glucose (pH = 7.4). Suspended cells were placed on the NPC© chip surface, and the whole cell configuration was achieved. Internal recording solution containing (in mM): 50 CsCl, 60 CsF, 10 NaCl, 10 HEPES, 2 Na^+^-ATP (pH = 7.2, adjusted with CsOH) was deposited in recording chips, having resistances of 3–5 MΩ. SOCs were activated with thapsigargin included in the intracellular solution. Physiological Ca^2+^ buffering was emulated using 0.2 mM of EGTA in the intracellular solution, namely weak Ca^2+^ buffer. To maintain mitochondria in an energized status, weak Ca^2+^ buffer was supplemented with a cocktail containing 2 mM pyruvic acid, 2 mM malic acid, and 1mM NaH_2_PO_4_ [9]. In a set of experiments, strong Ca^2+^ buffer internal solution containing 20 mM of EGTA was used. SOCs were assessed using voltage ramps (–100 to + 100 mV in 200 ms) applied every 5 s, from a holding potential of 0 mV and acquired with an EPC-10 patch-clamp amplifier (HEKA). Immediately after the whole-cell configuration was established, the cell capacitance and the series resistances (< 10 MΩ) were measured. During records, these two parameters were measured, and if exceed ≥ 10% respect to the initial value, the experiment was discontinued. Leak currents were eliminated by subtracting the average of the first five ramp currents (obtained just after whole cell configuration was reached) to all subsequent currents. Inward and outward current amplitudes were measured at –80 mV and +80 mV, respectively. Data were normalized respect to cell capacitance. Liquid junction potential and capacitive currents were cancelled using the automatic compensation of the EPC-10. Data were filtered at 10 kHz and sampled at 5 kHz.

### Bioluminescence imaging of mitochondrial [Ca^2+^]

Cells were transfected with the mitGA plasmid [25] using a Nucleofector II device (Amaxa Biosystems, Cologne, Germany) as reported previously [19, 20]. This plasmids contain wild type aequorin targeted to mitochondria and a GFP sequence to select transfected neurons for bioluminescence imaging. After 24 h, cells were incubated for 2 h with 4 μM coelenterazine at room temperature. This incubation time is required for the reconstitution of aequorin enzyme, thus enabling Ca^2+^-dependent light emission. Then, cells were washed and placed into a perfusion chamber thermostated to 37°C under a Zeiss Axiovert S100 TV microscope. For bioluminescence imaging, cells were perfused at 5–10 ml/min with test solutions based on the standard perfusing solution described above prewarmed at 37°C. At the end of each experiment, cells were permeabilized with 0.1 mM digitonin in SEM containing 10 mM CaCl_2_, added here to release all the residual aequorin counts. Bioluminescence images were taken with a Hamamatsu VIM photon counting camera handled with an Argus-20 image processor. Photonic emissions were integrated for 10 s periods. Photons were quantified using the Hamamatsu Aquacosmos software. Photonic emissions were converted to mitochondria free Ca^2+^ concentration ([Ca^2+^]mit) values as reported previously [19, 20, 32].

### Mitochondrial potential and mitochondrial depolarization

Cells were loaded with TMRM (10 nM, 30 min) at room temperature and placed on a Zeiss Axiovert 100 TV inverted microscope. For comparison of ΔΨ in NCM460 and HT29 cells, cells were loaded in parallel and TMRM fluorescence images were captured using exactly the same imaging parameters. Then, cells were treated with FCCP 10 μM to collapse mitochondrial potential. After 10 min, a fluorescence image was taken to be used as background image. For monitoring effects of NSAIDs on ΔΨ, TRMR-loaded cells were subjected to fluorescence imaging at 5 s intervals. At the end of the experiment, cells were treated with FCCP 10 μM for 10 min before capturing a fluorescence image to be used as background fluorescence for cells. Fluorescence intensity from regions of interest corresponding to individual cells were average and the average value after collapse of the mitochondria potential with FCCP was substracted from each region of interest. Fluorescence values from individual cells were normalized (expressed as the percentage value of fluorescence before addition of NSAIDs) and averaged. To quantify mitochondrial depolarization, the effect of NSAIDs or FCCP on the ratio of TMRM fluorescence in mitochondria relative to that surrounding cytosol was calculated as reported previously [17, 18].

### Quantitative real-time PCR

Total cellular RNA was isolated from cells using Trizol reagent (Invitrogen, Carlbads, CA, USA). Extracted RNA integrity was tested by electrophoresis on agarose gels and the purity and concentration were determined by spectrophotometry. RNA was reverse transcribed using a High Capacity cDNA Reverse Transcription Kit (Applied Biosystems, Foster City, CA, USA) and the cDNA diluted prior to PCR amplification. Primers used are shown in Table 1 and were designed using Primer-BLAST except for β-actin oligonucleotides that were taken from ref. 15. Real-time, quantitative PCR was performed using a SYBR green I detection in a LightCycler rapid thermal cycler (Roche, Mannheim, Germany). The PCR protocol started with 5 min at 95°C followed by 45 cycles of 15 s at 95°C, 20 s at 57°C or at 60 °C and 5 s at 72°C. β-actin was used as housekeeping gene. The data were normalized by PCR analysis of β-actin. Melting curves were used to determine the specificity of PCR products (Data not shown).

**Table 1:** Primers used for PCR experiments

Name	Primers (5→3)	Predicted size (pb)
MCU	F: TCCTGGCAGAATTTGGGAGC R: GGTGGTCGTACGTGGTATGT	320
MICU1	F: CTCGCAGCCTCCCTAAGATG R: GGAGACAGGGAACTTTGGGG	215
β-actin	F: TACGCCAACACAGTGCTGTCTGG R: TACTCCTGCTTGCTGATCCACAT	206

MCU and MICU1 primers were designed using BLAST-primer software and â-actin primers were taken from ref. 15. F: forward. R: reverse.

### Western blotting

Total protein was extracted from cells and used to quantify expression of MCU. Whole-cell lysate was obtained using RIPA buffer (20 mM Tris–HCl, pH 7.8, 150 mM NaCl, 1% Triton X-100, 1% deoxycholic acid, 1 mM EDTA, 0.05% SDS) supplemented with the Halt™ Protease and Phosphatase Inhibitor Cocktail (100X) from ThermoFisher Scientific (ref #1861281) (Waltham, MC, USA). Protein concentrations were determined by a Bradford protein assay. Proteins were fractionated by SDS-PAGE, electroblotted onto PVDF membranes and probed with the antibodies at dilution 1/200 except the anti-β-actin that was used at dilution 1/5000. The antibody against MCU (SC-246071) has been previously characterized [45] was visualized by addition of goat anti-rabbit IgG or rabbit anti-mouse IgG. Detection was performed using Pierce ECL Western Blotting substrate (Thermo Scientific) and VersaDoc Imaging System (BioRad, Munich, Germany). Quantification of protein expression was carried out using Quantity One software (BioRad, Munich, Germany).

### Statistics

When only 2 means were compared, student's t test was used. For more than 2 groups, statistical significance was assessed by ANOVA and compared using Bonferroni's multiple comparison tests. Differences were considered significant at *p* < 0.05.
